# ADMGCN: graph convolutional network for Alzheimer’s disease diagnosis with a meta-learning paradigm

**DOI:** 10.1093/bioinformatics/btaf580

**Published:** 2025-10-28

**Authors:** Xiaowen Sun, Jiahao Li, Guiying Yan, Renmin Han

**Affiliations:** College of Medical Information and Engineering, Ningxia Medical University, Yinchuan 750004, China; Research Center for Mathematics and Interdisciplinary Sciences (Ministry of Education Frontiers Science Center for Nonlinear Expectations), Shandong University, Qingdao 266237, China; Research Center for Mathematics and Interdisciplinary Sciences (Ministry of Education Frontiers Science Center for Nonlinear Expectations), Shandong University, Qingdao 266237, China; Academy of Mathematics and Systems Science, Chinese Academy of Sciences, Beijing, China; School of Mathematical Sciences, University of Chinese Academy of Sciences, Beijing 100190, China; College of Medical Information and Engineering, Ningxia Medical University, Yinchuan 750004, China; Research Center for Mathematics and Interdisciplinary Sciences (Ministry of Education Frontiers Science Center for Nonlinear Expectations), Shandong University, Qingdao 266237, China; Syneron Opal, 10281, Cayman Island

## Abstract

**Motivation:**

Alzheimer’s disease (AD) is a neurodegenerative disorder characterized by memory loss and cognitive decline. While graph convolutional networks (GCNs) have emerged as popular tools for AD diagnosis due to their ability to handle structural information and fuse multi-modal features, deep learning approaches face significant challenges including the requirement for large datasets and sensitivity to unbalanced label distributions in AD research. To address these limitations and enhance the flexibility of GCNs, we propose a graph convolutional network based on the meta-learning paradigm (ADMGCN) for early AD diagnosis. This approach incorporates weighting and dimensionality reduction to improve performance, storage, and training efficiency. By leveraging meta-learning, we sample subjects to create numerous label-balanced tasks, maximizing data utilization and mitigating the impact of label imbalance. Additionally, the meta-learning framework enables rapid adaptation to new tasks and facilitates independent testing of the GCN.

**Results:**

Our model, ADMGCN, was extensively validated on the Alzheimer’s Disease Neuroimaging Initiative datasets. It achieved a maximum accuracy of 73.7% in the multi-classification task for early AD diagnosis. In three binary classification tasks, the model also demonstrated strong performance, achieving accuracies of 92.8%, 88.0%, and 79.6%, respectively. These results confirm that the proposed method provides an effective approach and worthwhile support for the early diagnosis of Alzheimer’s disease.

**Availability and implementation:**

ADMGCN is freely available at https://github.com/WendySun16/ADMGCN.

## 1 Introduction

Alzheimer’s disease (AD) is a neurodegenerative disease, caused by damage to nerve cells in the brain, and it is the most common cause of dementia (Alzheimer’s Association 2023). AD has become one of the most disabling and burdensome diseases worldwide (Ferri *et al.* 2005), which is a continuous process from patients’ inability to detect problems in the brain at the beginning to eventually causing various disorders in the body due to the influence of the brain. The whole process is broadly segmented into three stages: normal cognition (NC), mild cognitive impairment (MCI), and Alzheimer’s disease (AD) (Shaw *et al.* 2009, Suk *et al.* 2015). At present, the treatment of Alzheimer’s disease remains challenging, and early intervention can help alleviate its adverse effects. Therefore, the early diagnosis of Alzheimer’s disease has become a topic of great significance (Li *et al.* 2023).

Functional magnetic resonance imaging (fMRI), particularly resting-state fMRI (rs-fMRI), serves as a critical diagnostic tool for Alzheimer’s disease research by capturing intrinsic blood-oxygen-level-dependent (BOLD) signals that reveal functional connectivity patterns between brain regions. These signals enable the construction of brain functional networks that facilitate early AD detection and analysis. Within this domain, graph neural networks (GNNs) have emerged as particularly powerful deep learning frameworks, leveraging their structural modeling capabilities to integrate both image-derived functional connectivity data and non-imaging biomarkers. Recent GNN advancements (Li *et al.* 2023, Zhou *et al.* 2023, Zuo and Kamata 2023) demonstrate significant potential for improving AD diagnostic accuracy through novel architectures.

Deep learning excels at discovering data structures but requires large, balanced datasets, presenting significant challenges in Alzheimer’s disease (AD) research due to the scarcity of rs-fMRI images, small cohort sizes, frequent label imbalance, and the cumbersome preprocessing pipeline. Graph Neural Networks (GNNs) face additional inflexibility, requiring retraining for new subjects. Meta-learning (Tian *et al.* 2022) emerges as a promising solution, leveraging prior knowledge and task-based learning to mitigate data scarcity issues. Crucially, it enables effective adaptation of GNNs for few-shot learning and circumvents the inflexibility of traditional GNNs by facilitating testing on new subjects without full retraining. However, the application of meta-learning, particularly combined with GNNs, to AD diagnosis remains underexplored, with challenges like limited data scale often unaddressed.

Among the approaches to studying AD diagnosis through the application of deep learning, graph neural networks are popular due to their strong structural capabilities and ability to integrate image and non-image data (Parisot *et al.* 2018). Notable works include Li *et al.*’s dual interpretable graph convolutional network for AD diagnosis (Li *et al.* 2023), Zhu *et al.*’s interpretable dynamic graph convolutional network for flexible graph structures (Zhu *et al.* 2022), Subaramya *et al.*’s integration of graph convolutional networks with traditional convolutional layers for AD classification (Subaramya *et al.* 2022), Zuo *et al.*’s attention-based model using 3D hypergraph convolutional networks (Zuo and Kamata 2023), Zhou *et al.*’s multi-modal imaging and genetic graph convolution framework for AD-related clinical score regression (Zhou *et al.* 2023), and Lei *et al.*’s multi-scale enhanced graph convolutional network for MCI detection (Lei *et al.* 2023).

To overcome the above difficulties, we propose a graph convolution model (ADMGCN) based on meta-learning paradigm in this paper. In the following, we regard each “subject” as a “node.” The model uses the graph convolutional network in the meta-learning mode to classify nodes for early diagnosis of AD. The functional connection information provided by rs-fMRI is utilized as the node features of the graphs through the attention mechanism and dimensionality reduction process. And the non-image information is combined to form small graphs as input to graph convolutional networks.

The three main contributions of this article are summarized as follows:

- A novel model based on a meta-learning paradigm is applied to early diagnosis of AD, thus solving the problem of limited data well. In addition, the trouble of uneven distribution of data labels has also been alleviated to a certain extent.
- Our model assigns weight to the functional connections of brain regions, which aids in understand the importance of each functional connection in the early diagnosis of AD.
- We make reasonable use of graph convolutional networks, which have excellent structure and the advantage of combining image information and non-image information. At the same time, the difficulty that new subjects are unable to be tested independently is worked out by constructing small graphs based on the meta-learning paradigm.

## 2 Materials and methods

### 2.1 Dataset

This study utilized resting-state fMRI (rs-fMRI) data from the Alzheimer’s Disease Neuroimaging Initiative (ADNI) database, analyzing cohorts of Alzheimer’s Disease (AD, *n* = 30), Mild Cognitive Impairment (MCI, *n* = 92), and Normal Control (NC, *n* = 243) participants. Subject demographic (age, gender) and APOE genotype information was also collected. Raw rs-fMRI data underwent standard preprocessing (including removal of initial volumes, slice-timing correction, realignment, normalization, smoothing, detrending, nuisance regression—Friston 24-parameters—band-pass filtering, scrubbing) and static functional connectivity network construction (using the AAL90 or Craddock200 atlas) primarily using the GRETNA toolkit. Comprehensive details on participant selection criteria, demographic characteristics, APOE4 distribution, and specific preprocessing parameters are provided in the Supplementary Material.

### 2.2 Process overview

**Figure 1.**
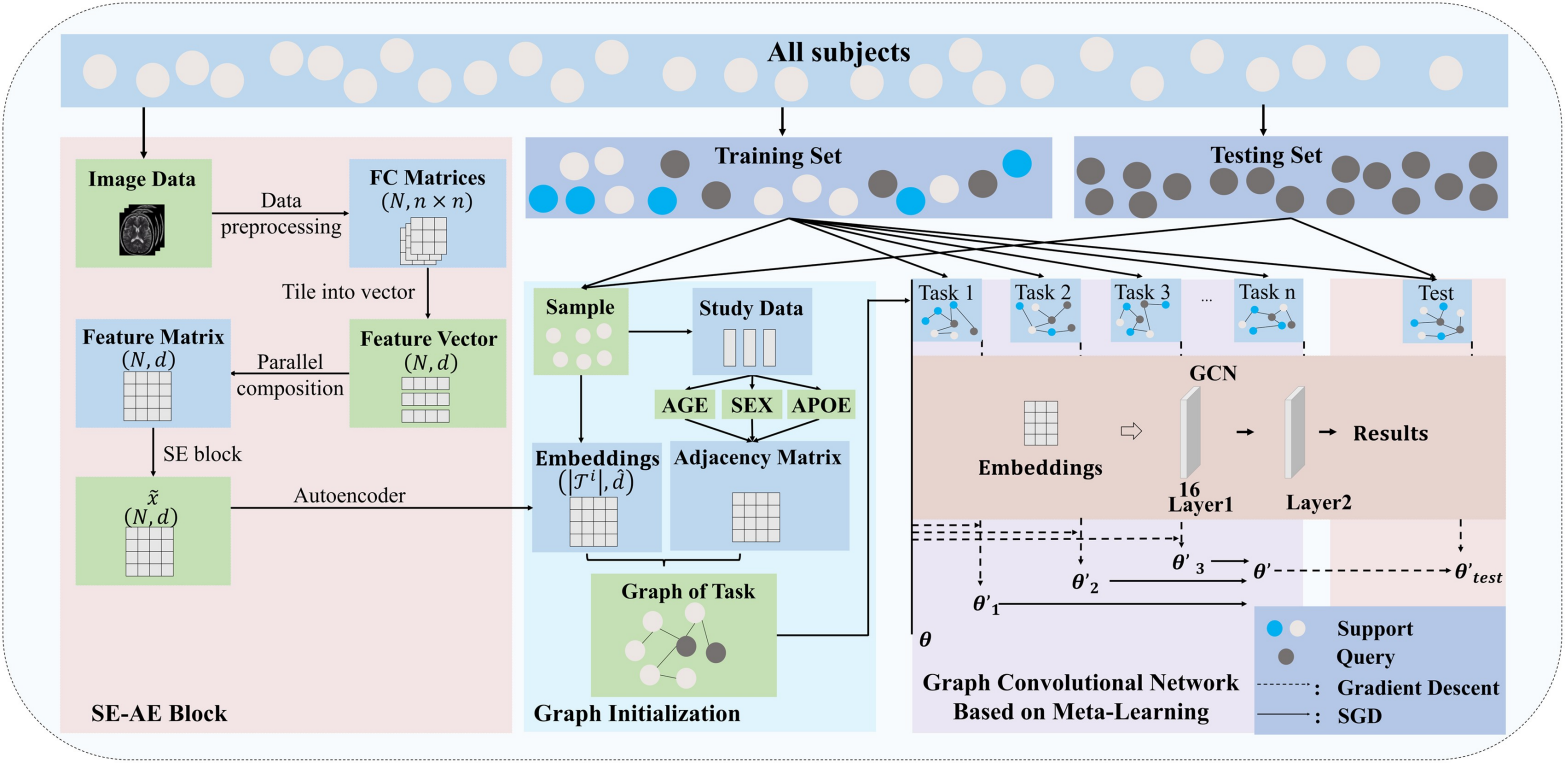
The pipeline of ADMGCN. ADMGCN takes the subjects as nodes and performs node classification tasks. The whole pipeline will go through (i) the feature processing stage, namely the SE-AE block (which processes raw features of dimension *d* into reduced-dimension features $\hat{d}$  ≪  *d* via Squeeze-Excitation weighting and Autoencoder compression), and (ii) the training and testing stages of graph convolutional network based on meta-learning. The meta-learning workflow includes: task sampling, support-set updates to obtain task-specific parameters (θ′), query-set loss for meta-gradient computation, global parameter update of *θ*, and meta-testing with one-step adaptation.

After obtaining the functional connectivity matrices of the brain regions by GRETNA, the whole pipeline of our study consists of two parts, as depicted in Fig. 1.

#### 2.2.1 Feature processing

Before the model is formally trained, the processing of features is essential. We implement this with a SE-AE block. SE-AE block has the ability to weight and dimensionally reduce the dimension of the original features, which also enables us to obtain effective information from the features. Moreover, the performance and efficiency of the model are improved (see Section 2.3 for details).

#### 2.2.2 Model training

We apply the graph convolutional network based on meta-learning to the early diagnosis of AD. In the meta-learning paradigm, the small graphs are initialized to form the basic unit of meta-learning, i.e. tasks. Then, we can train and evaluate the model through the meta-training stage and the meta-testing stage. During the training stage, each graph passes through a two-layer graph convolutional network, and the 2-fold gradient descent is utilized to obtain the most potential parameters. During the testing stage, the testing set is predicted brilliantly after fine-tuning the model (see Section 2.4 for details).

### 2.3 SE-AE block

In this section, we process the features of subjects to adapt them for input into a graph convolutional network, thereby improving training efficiency and model performance. After the pretreatment as above, each subject gets the corresponding functional connectivity matrix(*n*-order). Specifically, we flatten the upper triangular part of the functional connectivity matrix into a vector, denoted as $x_{i}=[x_{i}^{1},x_{i}^{2},\ldots ,x_{i}^{d}]\in R^{d},i=1,2,\ldots ,N,$ where *d* is the dimension of the feature. Each element of the feature represents the functional connection between brain regions. *N* is the number of subjects participating in the experiment.

#### 2.3.1 Squeeze-and-Excitation (SE)

**Figure 2.**
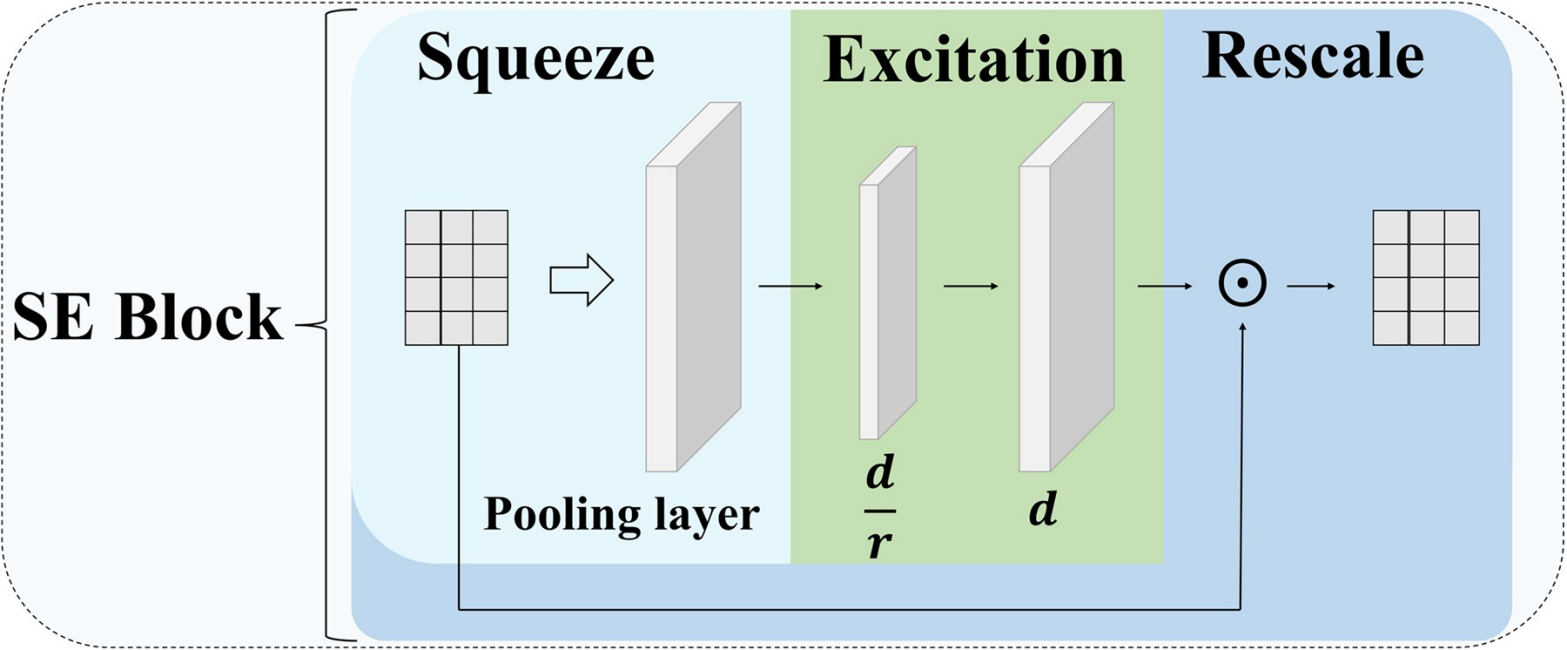
Architecture diagram of SE model. This model consists of three parts: Squeeze, Excitation, and Rescale, where ⊙ represents the element wise multiplication of two matrices.

To strengthen the understanding of the importance of functional connectivity between brain regions, we introduce SE block, inspired by SENet (Hu *et al.* 2018). This block consists of three parts, as illustrated in Fig. 2.

##### 2.3.1.1 Squeeze

We expect to use global subject information to explain and analyze the importance of functional connections between brain regions. Therefore, we aggregate the features of all subjects. We choose to perform a global average with vectors, considering that each subject has the same contribution, and obtain a global vector representation of functional connections between brain regions, denoted as $z=[z_{1},z_{2},\ldots ,z_{d}]\in R^{d}$. The vector representing each element of z can be obtained by the following calculation:


(1) $$z_{j}=\frac{1}{N}\sum_{i=1}^{N}x_{i}^{j},$$

where j=1,2,…,d represents each dimension of the features, with a total of *d* dimensions.

##### 2.3.1.2 Excitation

After aggregating global information, we need sufficient nonlinear interaction between the functional connections of brain regions to fully mine the relational information, so the global vector representation is first compressed. To obtain the importance of each functional connection, i.e. the weight information, we then try to reconstruct the compressed vector to the *d*-dimensional vector $s=[s_{1},s_{2},\ldots ,s_{d}]\in R^{d}$, where s is the weight of each functional connection between brain regions that we need. To limit the complexity of the model and improve the computational efficiency, we use two fully connected layers to achieve the above goals. The global vector first passes through a dimensionality-reduction layer with a rate of r, an activation function ReLU, then returns a *d*-dimensional vector through a dimensionality-increasing layer and finally passes through the activation function sigmoid. The whole process can be expressed as:


(2) $$s=\sigma \left(W_{2}\delta \left(W_{1}z^{T}\right)\right),$$

where σ is sigmoid, δ is ReLU, $W_{1}\in R^{\frac{d}{r}\times d},W_{2}\in R^{d\times \frac{d}{r}}$.

##### 2.3.1.3 Rescale

After obtaining the importance of each functional connection, we then assign the importance value to the original feature vector of each subject, i.e. weighted processing, to enhance the influence of important functional connections. Therefore, subsequent early Alzheimer’s disease diagnosis task can capture feature information more effectively:


(3) $${\tilde{x}}_{i}=s⊙x_{i}\;,$$

where ⊙ is the weight vector s multiplied by the original feature of the *i*th subject element by element to obtain the latest feature representation ${\tilde{x}}_{i}$ of the *i*th subject, which highlights the functional connections of important brain regions. By using the SE block, we facilitate the interaction of functional connection information, emphasizing connections that significantly impact diagnosis while downplaying those with lesser influence. Thus, this block helps enhance the model’s performance.

#### 2.3.2 Autoencoder (AE)

**Figure 3.**
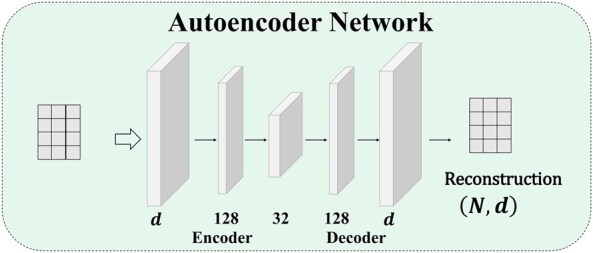
Network architecture diagram of autoencoder. The autoencoder structure is divided into two parts: encoder and decoder, each consisting of two neural network layers.

Each feature is a *d*-dimensional vector, which is a very high-dimensional vector because it is tiled by the upper triangular elements of the functional connection matrix. A very high feature dimension will lead to a series of problems, such as inefficiency in data storage, calculation, model training, etc. Therefore, we apply the Autoencoder (Bank *et al.* 2023) block to realize the dimensionality reduction of high-dimensional feature vectors, and it is also the process of extracting the most refined feature representation. This block helps to boost the model’s performance and efficiency. The network architecture is shown in Fig. 3.

##### 2.3.2.1 Unsupervised learning

To perform effective and reasonable dimensionality reduction on our existing features, we need to train an Autoencoder that is suitable for functional connectivity of brain regions. The training process requires training an encoder and a decoder, which is an unsupervised training process. First, the training set of features, which were obtained by the SE block, inputs into the encoder for dimensionality reduction. This process is completed through two layers of fully connected layers. Then the dimensionality reduction vectors are also returned to the original dimension through two fully connected layers, which constitute the decoder. The network is trained by comparing the returned vector with the original vector, and the model with good performance of the validation set is selected as the final model to ensure that the model has good generalization ability.

##### 2.3.2.2 Feature dimension reduction

This operation is to input the feature vector $\tilde{x}={\left[{\tilde{x}}_{1},{\tilde{x}}_{2},\ldots ,{\tilde{x}}_{N}\right]}^{T}\in R^{N\times d}$ obtained by SE block into the previously trained Autoencoder, to get the feature vector $\hat{x}={\left[\hat{x_{1}},\hat{x_{2}},\ldots ,\hat{x_{N}}\right]}^{T}\in R^{N\times \hat{d}}$ after dimension reduction, where $\hat{d}$ is the dimension of the feature vector after dimensionality reduction and $\hat{d}\ll d$. The feature vectors can still represent the original feature information well and greatly improve the efficiency of data storage and model training.

### 2.4 Graph convolutional network based on meta-learning

In our study, the early AD diagnosis task is modeled as a subject-based node classification problem, and a meta-learning paradigm is adopted to strengthen the use of subject data and improve the problem of uneven distribution of subjects as much as possible. Graph convolutional network also has a outstanding structure. We integrate both image information and non-image information through GCN. The whole process includes a meta-training stage and a meta-test stage, shown in Fig. 1. In each stage, we randomly sample subjects from the corresponding subject sets to build small graphs, respectively as meta-training tasks and meta-testing tasks. The corresponding training set and test set in the meta-task are called the support set and query set. We adopt the model-agnostic meta-learning (MAML) paradigm (Finn *et al.* 2017), implemented using the PyTorch framework (available at: https://github.com/dragen1860/MAML-Pytorch), for its compatibility with GCN backbones and proven effectiveness in few-shot learning scenarios involving data scarcity and label imbalance. Therefore, MAML can be effectively combined with graph convolutional networks (Zhou *et al.* 2019). In the meta-training stage, we use double gradient descent for optimization in the meta-training tasks. The goal is to learn the initialization parameters with the most potential, so that the model can quickly adapt to new early AD diagnosis tasks and make the best classification prediction. In the meta-testing stage, our model has gained a good ability to handle new tasks. The model uses the support set of meta-testing tasks to fine-tune quickly and then makes classification predictions on the query set of tasks to measure the performance of the model. This section will fully elaborate on the training and testing process of the model based on the meta-learning strategy, so the following parts of this section will be composed of Graph initialization, Meta-training, and Meta-testing.

#### 2.4.1 Graph initialization

We denote the model as $f_{\theta }$ with parameters θ. The training set is denoted as $D_{\mathrm{train}}=\left\{\left({\hat{x}}_{1},y_{1}),({\hat{x}}_{2},\;y_{2}),\ldots ,({\hat{x}}_{N^{\prime }},\;y_{N^{\prime }}\right)\right\}$, where $y_{i}\in \left\{1,\ldots ,C\right\},i=1,2,\ldots ,N^{\prime },N^{\prime }<N$, i.e. $y_{i}$ represents the subject label and *C* is the number of classification categories. A meta-task is a small graph composed of only a few subjects, and its construction process is shown in Fig. 1. In the meta-training stage, we randomly sample from $D_{\mathrm{train}}$ to constitute *M* meta-training tasks, and the subject sets between tasks are allowed to intersect. Permitting resampling can effectively mitigate the challenge of limited data availability. Meta-training tasks are denoted as $T_{\mathrm{train}}=\left\{T_{\mathrm{train}}^{1},T_{\mathrm{train}}^{2},\ldots ,T_{\mathrm{train}}^{M}\right\}$, for each task $T_{\mathrm{train}}^{i},i=1,2,\ldots ,M,$ whose support set and query set are $S_{\mathrm{train}}^{i}$ and $Q_{\mathrm{train}}^{i}$, respectively, and $T_{\mathrm{train}}^{i}=S_{\mathrm{train}}^{i}+Q_{\mathrm{train}}^{i}$. The composition of the support set of the meta-training task is similar to that of the query set, which is composed of randomly sampling the same number of subjects from each classification category, namely $|S_{\mathrm{train}}^{i}|=Ck_{\mathrm{train}}^{s}$, $|Q_{\mathrm{train}}^{i}|=Ck_{\mathrm{train}}^{q},$ where $k_{\mathrm{train}}^{s}$ and $k_{\mathrm{train}}^{q}$ represent the number of subjects for each category in the support set and the query set, respectively. This approach allows us to achieve balanced labels across tasks, addressing label imbalance. In the meta-testing stage, we denote the testing set as $D_{\mathrm{test}}$, the subject labels in the testing set are unknown, and our model is required to make predictions. So for meta-testing tasks $T_{\mathrm{test}}=\left\{T_{\mathrm{test}}^{1},\;T_{\mathrm{test}}^{2},\ldots ,T_{\mathrm{test}}^{P}\right\}$, *P* is the number of meta-testing tasks, and its support set is composed of random sampling from the training set $D_{\mathrm{train}}$, also $|S_{\mathrm{test}}^{i}|=Ck_{\mathrm{test}}^{s},i=1,2,\ldots ,P$, and the subjects of query set are sampled from testing set $D_{\mathrm{test}}$. The number of subjects in the query set can be one or several. Now we do not distinguish the subject categories because the labels are unknown, and the subject sets between query sets of meta-testing tasks do not cross until all testing set subjects are sampled. We have $|Q_{\mathrm{test}}^{i}|=t$, then $P=\left\lceil \frac{\left|D_{\mathrm{test}}\right|}{t}\right\rceil$.

The above subject sampling methods constitute the vertex set of each small graph, and the node features are the feature vectors obtained by the SE-AE block because of their richness and importance. The edge set of the small graph was obtained by using the non-image features of the subjects, namely the gender, age, and APOE4 gene information. The more similar the non-image features between the subjects, the greater the correlation between the subjects, and the more necessary the edge connection. Adjacency matrix A quantifies subject-subject associations using non-imaging features (age/sex/APOE) in task-specific graphs containing support/query sets. We denote the adjacency matrix of the small graph as $A\in R^{n\times n}$, where *n* represents the number of nodes of the corresponding small graph, which is generated by the following formulas:


(4) $$a_{i,j}^{u}=\{\begin{array}{c} 1,1-\frac{\left|s_{i}^{u}-s_{j}^{u}\right|}{\mathrm{rang}e^{u}}\geq \beta_{1}\\ 0,\text{otherwise} \end{array},$$

(5) $$a_{i,j}=\{\begin{array}{c} 1,\frac{1}{3+1-\sum_{k}^{3}a_{i,j}^{u}}\geq \beta_{2}\\ 0,\text{otherwise} \end{array},$$

where the above two formulas are the case of i≠j, when $i=j,a_{i,j}=0$. $a_{i,j}$ represents the element of *i*th row and *j*th column in matrix *A*. $s_{i}^{u}$ represents the value of the *u*th non-image feature of the *i*th subject. $\mathrm{rang}e^{u}$ is the extreme value of the *u*th non-image feature of all subjects. Then $a_{i,j}^{u}$ describes the degree of similarity between the *i*th subject and *j*th subject on the *u*th non-image feature, where u=1,2,3. $\beta_{1}$ and $\beta_{2}$ are thresholds.

#### 2.4.2 Meta-training

In the meta-training stage, we expect to learn the initialization parameters that have the greatest potential for early diagnosis of AD. When facing new meta-tasks, these parameters can be quickly updated through a few times of gradient descent to get the best parameters of the corresponding tasks, and to make classification prediction more accurate.

The support set $S_{\mathrm{train}}^{i}$ of task $T_{\mathrm{train}}^{i}$ is fed to an *L*-layer graph convolution network as input first. Let ${\hat{X}}^{\left(0\right)}={\left[{\hat{x}}_{1},{\hat{x}}_{2},\ldots ,{\hat{x}}_{N^{\prime \prime }}\right]}^{T}\in R^{N^{\prime \prime }\times d}$ represent the input, where $N^{\prime \prime }=|S_{\mathrm{train}}^{i}|+|Q_{\mathrm{train}}^{i}|$. Then after l+1 layers, feature embedding ${\hat{X}}^{\left(l+1\right)}$ is obtained by the following:


(6) $${\hat{X}}^{\left(l+1\right)}=\delta \left({\tilde{D}}^{-\frac{1}{2}}\tilde{A}{\tilde{D}}^{-\frac{1}{2}}{\hat{X}}^{\left(l\right)}\theta^{\left(l\right)}\right),$$

where l=0,1,…,L−1, $\tilde{A}=A+I$. $\tilde{D}$ is a degree matrix of $\tilde{A}$. δ refers to an activation function.

After that, the cross-entropy loss function is used to calculate the loss:


(7) $$L_{T_{\mathrm{train}}^{i}}\left(f_{\theta }\right)=-\frac{1}{\left|S_{\mathrm{train}}^{i}\right|}\sum_{j}\sum_{c=1}^{C}y_{i_{j_{c}}}\;\text{log}\;f_{\theta }\left(x_{i_{j_{c}}}\right).$$

Using the above loss function and gradient descent method, we can update the model parameters θ in $T_{\mathrm{train}}^{i}$, and gradient descent can be updated one or more times. For simplicity and clarity, the following formula shows the one task-level inner gradient descent update process for $T_{\mathrm{train}}^{i}$:


(8) $$\theta^{\prime }{}_{i}=\theta -\alpha_{1}\nabla_{\theta }L_{T_{\mathrm{train}}^{i}}\left(f_{\theta }\right),$$

where $\alpha_{1}$ represents the step size of the local task, i.e. the learning rate. At this point, we get $\theta_{i}^{\prime }$, which is the preferred parameter for the current task $T_{\mathrm{train}}^{i}$. The purpose of our model is to update parameters θ to get parameters that perform well and have potential for all tasks, rather than just improving performance on the current task. Therefore, we need to collect the performance of query sets $Q_{\mathrm{train}}^{i}$ with $\theta_{i}^{\prime }$ of a batch of tasks to update the parameter θ via stochastic gradient descent. The specific process is as follows:


(9) $$\theta \leftarrow \theta -\alpha_{2}\nabla_{\theta }\sum_{J_{\mathrm{train}}^{i}∼P\left(J_{\mathrm{train}}\right)}L_{T_{\mathrm{train}}^{i}}\left(f_{\theta^{\prime }{}_{i}}\right),$$

where $P(T_{\mathrm{train}})$ represents the distribution of tasks and $\alpha_{2}$ is the meta-learning rate.

#### 2.4.3 Meta-testing

Through the above meta-training stage, we have obtained potential model parameters θ, which can be fine-tuned in the face of new tasks to achieve the best classification performance. Therefore, in the meta-testing stage, we input the meta-testing task $T_{\mathrm{test}}^{i}$ into the model and use the support set $S_{\mathrm{test}}^{i}$ of the task to perform one or several times of gradient descent. Then the optimal parameters $\theta_{\mathrm{test}}^{i}$ are obtained for the current task. Finally, the query set of the task is predicted to evaluate the model performance. Therefore, when one or several new subjects need to be predicted, we can control the size of the query set. Then the meta-learning process can easily perform independent testing.

By following the above series of processes, the purpose of predicting the testing set is achieved. The overall framework of ADMGCN can be referred to Algorithm 1.

Algorithm 1 Framework of ADMGCN

**Require:** Features of subjects: $x_{i},\;\;i=1,2,\ldots ,N$; Task-learning rate: $\alpha_{1}$; Meta-learning rate: $\alpha_{2}$.

**Ensure:** Labels for query sets of all $T_{\mathrm{test}}$

1:  Obtain $\hat{x}$ through the SE-AE block by all $x_{i}$.

2:  Form distribution over meta-training tasks $P(T_{\mathrm{train}})$ and over meta-testing tasks $P(T_{\mathrm{test}})$ by sampling

3:  Initialize θ randomly

4:  **while** not done **do** 

5:    Sample batch of meta-training tasks $T_{\mathrm{train}}^{i}∼P\left(T_{\mathrm{train}}\right)$

6:    **for** all $T_{\mathrm{train}}^{i}$  **do** 

7:      Calculate $L_{T_{\mathrm{train}}^{i}}\left(f_{\theta }\right)$ using $S_{\mathrm{train}}^{i}$ by (7)

8:      Compute $\theta_{i}^{\prime }$ by (8)

9:      Calculate $L_{T_{\mathrm{train}}^{i}}\left(f_{\theta_{i}^{\prime }}\right)$ using $Q_{\mathrm{train}}^{i}$

10:    **end for** 

11:    Update θ by (9)

12:  **end while** 

13:  **for** all $T_{\mathrm{test}}^{i}∼P\left(T_{\mathrm{test}}\right)$  **do** 

14:    Fine-tune θ using $S_{\mathrm{test}}^{i}$

15:    Predict labels for the query set of $T_{\mathrm{test}}^{i}$

16:  **end for** 

## 3 Experiment

### 3.1 Experimental settings

In this section, we will introduce our computational efficiency and resource consumption, describing the hardware and environment configuration used in the experiment, as well as the specific settings of various parameters. The experiments were conducted on a computer equipped with an NVIDIA A100-PCI graphics card. In the meta-learning process, the hyperparameters were set as follows: the learning rate was 0.01, and was 0.003. To reduce overfitting, we applied Dropout technique with a dropout rate of 0.5. To ensure more stable performance evaluation of the model, we used 5-fold cross-validation.

In the experiments, we calculated accuracy, sensitivity, specificity, AUC, standard deviation, and F1-score to evaluate the model’s performance. Accuracy quantifies overall classification correctness, while sensitivity and specificity respectively measure the model’s ability to correctly identify positive and negative cases. The AUC evaluates the overall discrimination capacity across classification thresholds, and the F1-score balances precision and recall for robust performance assessment in datasets.

These hardware configurations, parameter settings, and performance metric calculations will help us evaluate the model’s performance and provide valuable information for Alzheimer’s disease classification and diagnosis.

### 3.2 Comparison with different models

To verify the superior performance of our model, we compared it with different models, including support vector machines (SVM) (Cortes and Vapnik 1995), multilayer perceptron (MLP) (Kruse *et al.* 2022), logistic regression (LR) (Hastie *et al.* 2009), GAT (Veličković *et al.* 2018), and GCN (Kipf and Welling 2017). These models are fundamental and commonly used in machine learning and deep learning. We performed 5-fold cross-validation experiments five times on all models. It is worth noting that, all baseline models (SVM/MLP/LR/GAT/GCN) use original functional connectivity features. Only ADMGCN incorporates SE-AE feature processing. And we trained and predicted with these models using data processed via the AAL atlas. For our model, we conducted multi-label classification experiments using data processed based on both the AAL atlas and CC200 atlas. Results showed that for the AAL atlas, the mean accuracy across five rounds of 5-fold cross-validation experiments was 0.727, compared to 0.702 for the CC200 atlas. Thus, our model achieved better performance with the AAL atlas, and we present those results in the tables and use AAL atlas for subsequent experiments. The comparison results are shown in Tables 1 and 2.

**Table 1.** Performance of models for AD versus NC And AD versus MCI And MCI versus NC classification tasks.

Methods	AD versus NC				
	ACC	SEN	SPE	AUC	F1-score
SVM	0.890±0.000	0.000±0.000	1.000±0.000	0.500±0.000	0.000±0.000
MLP	0.884±0.006	0.147±0.027	0.975±0.009	0.561±0.010	0.206±0.012
LR	0.886±0.000	0.133±0.000	0.980±0.000	0.556±0.000	0.177±0.000
GAT	0.879±0.005	0.067±0.021	0.979±0.006	0.523±0.010	0.104±0.027
GCN	0.859±0.062	0.960±0.080	0.040±0.080	0.566±0.000	0.904±0.075
Ours	0.928±0.004	0.538±0.040	0.983±0.006	0.759±0.028	0.960±0.002

	AD versus MCI				
	ACC	SEN	SPE	AUC	F1-score
SVM	0.754±0.000	0.000±0.000	1.000±0.000	0.500±0.000	0.000±0.000
MLP	0.728±0.014	0.193±0.033	0.903±0.027	0.548±0.008	0.240±0.039
LR	0.705±0.000	0.233±0.000	0.859±0.000	0.546±0.000	0.275±0.000
GAT	0.650±0.046	0.227±0.071	0.789±0.078	0.508±0.022	0.182±0.032
GCN	0.734±0.040	0.960±0.080	0.040±0.080	0.548±0.000	0.826±0.069
Ours	0.880±0.011	0.579±0.055	0.964±0.014	0.758±0.072	0.920±0.007

	MCI versus NC				
	ACC	SEN	SPE	AUC	F1-score
SVM	0.725±0.000	0.000±0.000	1.000±0.000	0.500±0.000	0.000±0.000
MLP	0.654±0.010	0.214±0.015	0.821±0.011	0.518±0.010	0.250±0.017
LR	0.672±0.000	0.229±0.000	0.839±0.000	0.534±0.000	0.278±0.000
GAT	0.654±0.035	0.204±0.062	0.826±0.070	0.515±0.014	0.183±0.042
GCN	0.654±0.067	0.160±0.150	0.840±0.150	0.492±0.000	0.069±0.065
Ours	0.796±0.009	0.360±0.051	0.959±0.011	0.665±0.034	0.872±0.004

Comparison	*P*-values (Ours versus Methods)				
	SVM	MLP	LR	GAT	GCN
AD versus NC	3.83E-05	1.22E-06	2.57E-05	2.24E-07	9.06E-02
AD versus MCI	2.31E-05	1.48E-07	6.27E-06	3.55E-04	1.22E-03
MCI versus NC	7.60E-05	1.87E-08	8.38E-06	9.24E-04	1.25E-02

**Table 2.** Performance of models for multi-label classification tasks.

Methods	AD versus MCI versus NC				
	Max	Min	Mean	Std	F1-score
SVM	0.666	0.666	0.666	0.000	0.266
MLP	0.633	0.586	0.613	0.017	0.362
LR	0.627	0.627	0.627	0.000	0.369
GAT	0.614	0.584	0.597	0.012	0.315
GCN	0.666	0.466	0.592	0.074	0.459
Ours	0.737	0.723	0.727	0.005	0.519
*t*-test	Ours versus Methods				
	SVM	MLP	LR	GAT	GCN
*P*-value	1.92E-05	6.39E-05	2.68E-06	4.91E-08	2.12E-02

For binary classification, the ACC of our model reached 0.928, 0.880 and 0.796, respectively, which were superior to other models, and our AUC and F1-score were also optimal. In addition, it is worth noting that we can clearly see from the SEN and SPE that other models were severely affected by the unbalanced data distribution, especially the SVM and GCN models. Our model obviously ameliorated the effect of this imbalance, which can also show the excellent performance of our model.

For multi-label classification, our model achieved a mean accuracy of 0.727, with the maximum accuracy reaching 0.737, significantly surpassing other models. This revealed that our model also had excellent performance on multi-label classification task. And our model outperformed others in terms of F1-score. Moreover, the *P*-value from the t-test demonstrated that our model’s accuracy was significantly higher than that of the baseline models.

Meanwhile, in terms of multi-label classification, we compared the performance of our model with other existing works, as shown in Table 3. Our model achieved an accuracy of 0.737 using only rs-fMRI image data and non-image features, which was excellent among other models. However, from the columns of data types and subjects in the table, we can find that there were differences in the data types, subject-sizes, and subject-distributions used in each work. Therefore, the comparison between models provided only a partial reference. Please note that the comparative method used in this study is also adopted by Wang *et al.* (2020) and others, and it is a commonly used approach in the field of early diagnosis of Alzheimer’s disease.

**Table 3.** Performance comparison of different methods reported in the references.^a^

AD versus MCI versus NC			
References	Data types	Subjects	ACC
Wang *et al.* (2020)	rs-fMRI	99AD/310MCI/154NC	0.718
Tong *et al.* (2017)	CSF/MRI/PET	37AD/75MCI/35NC	0.602
Zuo and Kamata (2023)	fMRI	16AD/26MCI/30NC	0.714
Itkyal *et al.* (2023)	sMRI/rs-fMRI	83AD/264MCI/383NC	0.627
Lin *et al.* (2021)	MRI/PET/CSF/gene	105AD/441MCI/200NC	0.667
Ours (Mean)	rs-fMRI	30AD/92MCI/243NC	0.727
Ours (Max)	rs-fMRI	30AD/92MCI/243NC	0.737

a Results are provided as a broad reference across modalities; direct comparison is not valid due to variations in data types, cohort sizes, and processing pipelines.

### 3.3 Ablation experiment

We conducted ablation experiments to evaluate the contribution of each component in our ADMGCN model, with results presented in Table 4. The experiments utilized a single 5-fold cross-validation setup, where ADMGCN represented the complete model, while -SE, -AE, -Meta, -NI, and -SE-AE denoted versions with the SE block, Autoencoder block, meta-learning strategy, non-image features, or both SE and Autoencoder blocks removed, respectively. We also reported per-iteration training time and sensitivity to subject-label imbalance, marked by “√” for high sensitivity and “×” for low. The results clearly showed that ADMGCN, incorporating all modules, achieved the highest F1-score with low standard deviation, indicating superior performance and predictive consistency. Notably, models without the Autoencoder block (-AE and -SE-AE) required significantly longer training times, highlighting the Autoencoder’s role in reducing feature dimensions and enhancing training efficiency. Additionally, -AE, -Meta, and -SE-AE were more affected by subject-label imbalance, underscoring the importance of the Autoencoder and meta-learning strategy in mitigating this issue by transforming high-dimensional features into lower dimensions without information loss and ensuring label balance at the task level.

**Table 4.** Results of each model of ablation experiments.

Methods	AD versus MCI versus NC				Iteration time(s)	Impact of data imbalance
	ACC			F1-score		
	Max	Min	Mean			
−SE	0.794	0.616	0.726±0.067	0.539	3.569	×
−AE	0.726	0.575	0.671±0.053	0.299	13.990	√
−Meta	0.671	0.658	0.666±0.007	0.532	0.059	√
−NI	0.616	0.493	0.567±0.043	0.351	3.722	×
−SE-AE	0.699	0.562	0.655±0.048	0.288	21.075	√
ADMGCN	0.794	0.644	0.737±0.057	0.495	2.249	×

### 3.4 Influence of the graph size

**Figure 4.**
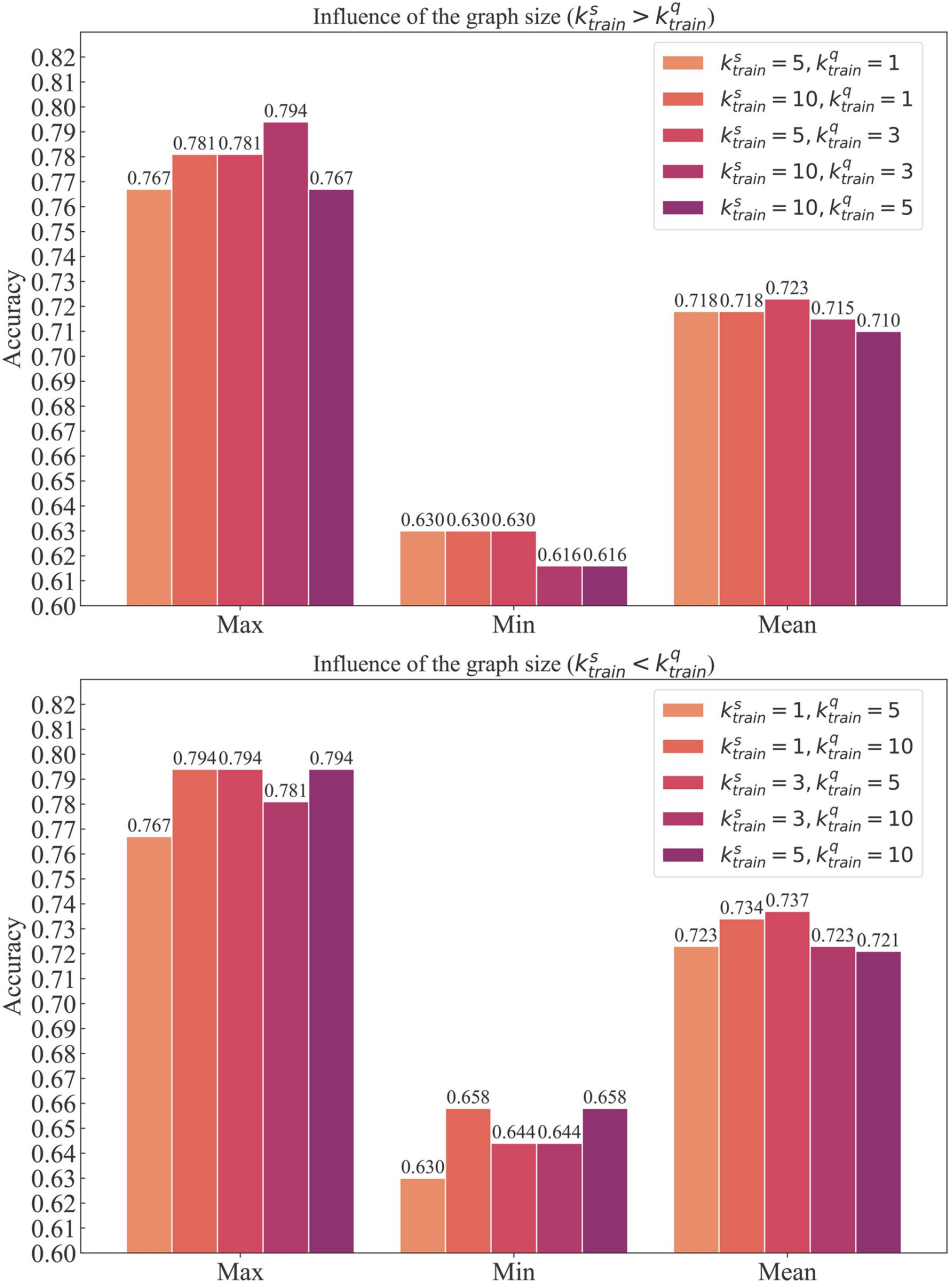
Performance of different graph sizes. We draw a bar chart of influence of the graph size in this figure with two different graphs: (a) $k_{\mathrm{train}}^{s}>k_{\mathrm{train}}^{q}$ and (b) $k_{\mathrm{train}}^{s}<k_{\mathrm{train}}^{q}$.

We explored whether the size of the small graph per task had a significant impact on performance. A small, simply structured graph enhanced model-training efficiency and more, but it was not worth it if model performance suffered. We adjusted the sizes of $k_{\mathrm{train}}^{s}$ and $k_{\mathrm{train}}^{q}$ to carry out multi-label classification experiments. Meanwhile, we divided into $k_{\mathrm{train}}^{s}>k_{\mathrm{train}}^{q}$ and $k_{\mathrm{train}}^{s}<k_{\mathrm{train}}^{q}$ two cases. The accuracy of the experiment in shown in Fig. 4.

The experimental results showed that the variation in graph size had little effect on the model’s performance. Even when using very small graphs for training, we can also receive impressive accuracy results. Among the graph scales we tried, $k_{\mathrm{train}}^{s}=3$, $k_{\mathrm{train}}^{q}=5$ gave the best result, indicating that a moderate graph size was optimal. Smaller graphs provided relatively less information, while larger graphs added complexity, which can affect model performance. Furthermore, the accuracy results for $k_{\mathrm{train}}^{s}<k_{\mathrm{train}}^{q}$ were generally better than those for $k_{\mathrm{train}}^{s}>k_{\mathrm{train}}^{q}$. Since the query set of each task is directly applied to update the model parameters θ, it is more beneficial to improve the performance of the whole model when the query sets have more subjects.

## 4 Conclusion

In this paper, we proposed a graph convolutional network based on meta-learning strategy. Our model is supplemented by SE, Autoencoder and GCN with the meta-learning framework. It effectively addresses the following limitations in AD research: limited AD research data, unbalanced data labels, and independent testing of GCN node classification. The SE module weights the features of the input neural network, giving greater influence to features that are more important to the task of early diagnosis of AD. The Autoencoder module improves the efficiency and performance of our models. GCN has the ability to use structural information for diagnosis. Meta-learning provides an effective solution to problems such as too small data volume, imbalanced sample labels and the inability of GCN to be tested independently. Through effective experimental verification, our model had good performance in the early diagnosis of Alzheimer’s disease, with a maximum accuracy of 0.737 through 5-fold cross-validation. What’s more, we also effectively verified the rationality and validity of each module of the model through ablation experiments. Hence, ADMGCN has great practical significance. On the other hand, this study can be further optimized in the future.

## Supplementary Material

btaf580_Supplementary_Data

## Data Availability

The data underlying this article were obtained from the Alzheimer’s Disease Neuroimaging Initiative (ADNI) database (adni.loni.usc.edu).
