# Supplementary material for: ADMGCN: graph convolutional network for Alzheimer’s disease diagnosis with a meta-learning paradigm
Source: Bioinformatics. 2025 Oct 28;41(12):btaf580. doi: 10.1093/bioinformatics/btaf580 (PMC12701806; doi:10.1093/bioinformatics/btaf580)
Supplement: btaf580_Supplementary_Data [file btaf580_supplementary_data.pdf]

Supplementary Materials for "ADMGCN: Graph Convolutional Network for Alzheimer's Disease Diagnosis with a Meta-learning Paradigm"

Xiaowen Sun[1,2,†], Jiahao Li[2,†], Guiying Yan[3,*], and Renmin Han[1,2,4,*]

[1]College of Medical Information and Engineering, Ningxia Medical University, Yinchuan 750004, China

[2]Research Center for Mathematics and Interdisciplinary Sciences (Ministry of Education Frontiers Science Center for Nonlinear Expectations), Shandong University, Qingdao 266237

[3]Academy of Mathematics and Systems Science, Chinese Academy of Sciences, Beijing, China and School of Mathematical Sciences, University of Chinese Academy of Sciences, Beijing, China, 100190

[4]Syneron Opal, 10281, Cayman Island

Dataset

In this study, we utilized the international Alzheimer's Disease Neuroimaging Initiative (ADNI) database (https://adni.loni.usc.edu) as our research material. The ADNI dataset was selected for its authoritative reputation, providing a robust and validated research foundation for the development of rs-fMRI-based algorithms. Specifically, we selected rs-fMRI data from the ADNI database for in-depth analysis. We carefully chose 30 subjects of AD patients, 92 subjects of MCI patients, and 243 subjects of NC individuals. These subjects represent different cognitive states and provide a valuable foundation for understanding the development of Alzheimer's disease.

Table 1: Summary OF The Subjects Used In This Study. The Age Presented In The Mean±Std Format.

Type	Number	Age	Gender (male/female)	APOE4 (0/1/2)
AD	30	76.5±7.9	20/10	12/10/8
MCI	92	73.9±8.6	52/40	53/31/9
NC	243	73.2±7.5	147/96	148/80/5

In addition to the rs-fMRI data, we also collected clinical information and features of the subjects, including age, gender, APOE gene information. These details are frequently used in early AD diagnosis research and help us extract features related to Alzheimer's disease more comprehensively.

During the data preprocessing stage, we employed the powerful tool called GRETNA (Graph Theoretical Network Analysis) [5]. GRETNA is widely used in Alzheimer's disease research for functional connectivity network analysis.

To ensure data quality and consistency, we conducted meticulous preprocessing of the rs-fMRI data. First, the first 10 time points were removed, and slice timing correction was performed with a TR (repetition time) of 3 seconds. Then, the data underwent realignment, normalization, and spatial smoothing. After that, linear detrending was applied to remove low-frequency drift, followed by regression of white matter signal, CSF signal, and head motion effects using the Friston 24-parameter model. Band-pass filtering and scrubbing were then

applied. Finally, static functional connectivity analysis was performed using the AAL90 [4] or Craddock200 atlas [2]. This process ensured the data was clean, aligned, normalized, and ready for further analysis.

Next, we applied GRETNA's functional connectivity analysis method to calculate the functional connectivity strength between each pair of brain regions. These functional connections can be represented as a connectivity matrix, where each element represents the connection strength between two brain regions. To visualize these data more intuitively, we used the Automated Anatomical Labeling (AAL) and Cameron Craddock's 200 ROI (CC200) as templates for functional connectivity network analysis. The AAL brain atlas divides the brain into 90 anatomically meaningful regions, allowing us to map the functional connectivity strengths to different pairs of brain regions. The CC200 brain atlas was proposed and developed by Craddock et al. in research, typically consisting of 200 nodes (or brain regions) and the connectivity information between them. It can be used to study the functional network structure of the brain, information transmission pathways, and the collaborative activities between different brain regions.

Experimental Environment

All computational experiments were conducted on a high-performance computing platform utilizing an Intel(R) Xeon(R) Platinum 8260 CPU @ 2.40GHz, accelerated by an NVIDIA A100-PCIE-40GB GPU with 1.0 TB of RAM. The system operated under Ubuntu 18.04.6 LTS with CUDA 11.2 and cuDNN 8.1.0 for GPU acceleration. Software implementations leveraged Python 3.10 and PyTorch 2.3.0, with additional dependencies including NumPy 1.23.5, scikit-learn 1.2.2, and PyTorch Geometric 2.3.0. This standardized environment ensures computational reproducibility across experimental phases.

Feature weighted analysis

We first obtained the connectivity weights between different brain regions using the SE block. Then, we selected the top 30 most important connections acquired by SE block and visualized them in **Figure 1**. with two different graphs: (a) a chord diagram and (b) a brain region map.

In both graphs, we only displayed the 43 brain regions out of the 90 regions from the AAL brain atlas that are involved in these 30 selected connections. The thickness of the lines in graph (a) represents the weight of the connections, with thicker lines indicating higher weights. On the other hand, graph (b) was generated by the BrainNet Viewer tool [6], utilizing the Interpolated Map algorithm. The color labels were arranged based on the weight ranking from 1 to 30.

We further investigated the functionality and significance of the brain regions associated with the onset of Alzheimer's disease. Yang et al.'s study identified differences in some brain regions [7]. They observed significant variations in the Left Hippocampus and Right Middle Frontal Orbital Gyrus regions between the Alzheimer's disease groups and the non-patient groups, indicating notable disparities in these areas during the progression of Alzheimer's disease. Luo et al.'s study found significant abnormalities in the Left Middle Frontal Gyrus region among Alzheimer's disease patients compared to non-patients, supporting previous research on the importance of this region in the disease [3]. The Right Middle Frontal Orbital Gyrus and Left Middle Frontal Gyrus regions, located in the frontal lobe, play a crucial role in cognitive processes due to their neural connections and structural patterns. And the Right Middle Temporal Pole region, responsible for auditory processing and linked to memory and emotions, also contributes to our understanding of Alzheimer's mechanisms. Kuljeet Singh Anand, Vikas Dhikav compiled a review [1], based on which we can learn that the hippocampus has a major role in learning and memory. Therefore, examining differences in the Left Middle Frontal Gyrus region and understanding the functional roles of prefrontal, temporal pole, and hippocampal regions provides insights into memory and cognitive decline in AD.

Our SE block identified meaningful pathways with higher weights in Alzheimer's disease. In our model, functional connections associated with the Left Middle Frontal Gyrus, Left Hippocampus, Right Middle Frontal

Orbital Gyrus, and Right Middle Temporal Pole also obtained relatively high weights. These connections were highlighted in the **Figure 1(a)**. Therefore, our model provided important insights into the study of abnormal brain regions and pathways to a certain extent. Further analysis of these pathways can deepen our understanding of the pathological mechanisms and offer accurate guidance for diagnosis and treatment.

Influence of gradient descent steps

We expected our model to adapt quickly when faced with new tasks, which meant performing well with only a few steps of fine-tuning. Furthermore, a small number of gradient descent steps help mitigate the over-smoothing issue in graph-related tasks. Therefore, we conducted experiments to verify the effect of adjusting the number of gradient descent steps on the support sets. The results are shown in **Figure 3**. We took the number of gradient descent as 1, 2, 3, 5, and 10 times respectively, and adopted the learning rate $\alpha_1 = 0.01$. We also fixed graph size with $k^s_{train} = 3$, $k^q_{train} = 5$. In addition, we use the same number of steps in both the meta-training stage and the meta-testing stage.

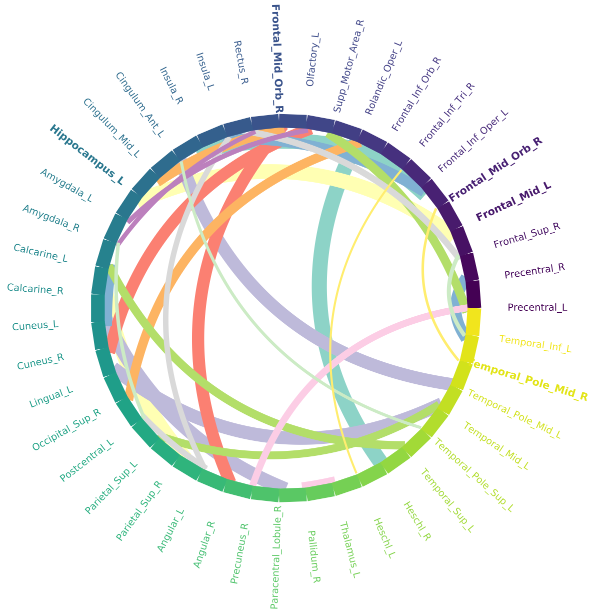

(a) Chord diagram

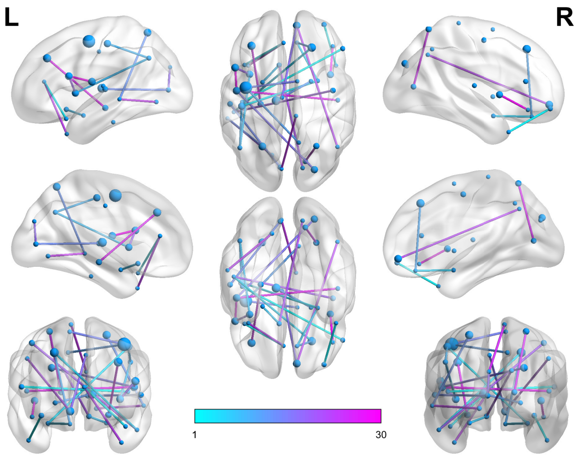

(b) Brain region map

Figure 1: Visualization of the top 30 functional connections. We selected the top 30 most important connections acquired by SE block and visualized them in Figure 1 with two different graphs: (a) a chord diagram and (b) a brain region map.

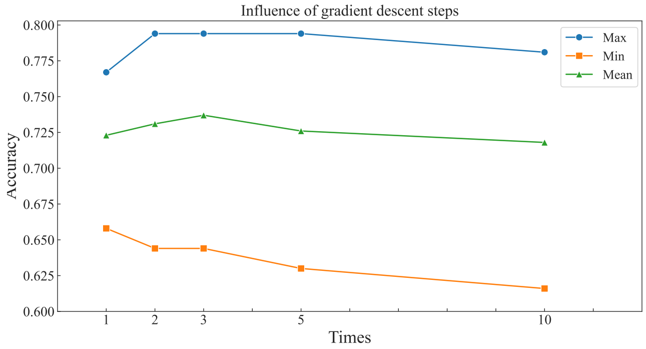

Figure 2: The model accuracy results of different gradient descent times on the support sets. We took the number of gradient descent as 1, 2, 3, 5, and 10 times respectively.

We were able to find from the results of the figure that the model can perform better in very few steps, or even only need one step gradient descent, which greatly improved the efficiency of model training and independent testing. The mean accuracy obtained by three times of gradient descent was the best. When only one step of gradient descent was acted, although its maximum value was weaker, it had the highest minimum value, indicating more stable performance. As the number of steps increased, the choice of learning rate became increasingly important. This could also be the reason why accuracy decreased with more steps and why the five-fold accuracy became increasingly unstable.

Dicussion

In recent years, deep learning has been widely used in the early diagnosis of Alzheimer's disease. However, the data we can use for this problem is limited, especially rs-fMRI, so it poses challenges for deep learning models that typically require large datasets. At the same time, the problem of label imbalance is also worthy of attention. Graph neural networks, commonly used to study AD problems, also have the disadvantage that it cannot be tested independently in node classification. Therefore, we proposed ADMGCN, which can effectively improve the above series of problems. Based on the meta-learning strategy, the model randomly sampled subjects to construct small graphs. The node features were weighted low-dimensional features processed by the SE-AE block. At the same time, the adjacency matrix was formed by non-image features. The small graphs were used as input to GCN for node classification, with the model's performance evaluated on the test set.

First, we tested the performance of model binary and multi-label classification, respectively. In the classification tasks of AD vs. NC, AD vs. MCI and MCI vs. NC, the accuracy of our model reached 0.928, 0.880 and 0.796. The SEN and SPE results also revealed that our model mitigated the effects of label imbalance. In the multi-label classification task, the model achieved an average accuracy of 0.727, with a peak of 0.737. The above results highlighted the excellent performance of our model. The meta-learning framework made us to expand the limited dataset by constructing numerous small graphs, which allowed subjects to overlap between graphs. For example, we originally had 30 subjects. The small graph needed to choose 8 subjects, then we can get $\binom{30}{8} = 5852925$ small graphs. This number is very large, which is more conducive to deep learning training. By ensuring an equal number of subjects from each label category in the construction process, we mitigated the impact of label imbalance. In addition, we achieved independent testing by controlling the query set size of the meta-testing tasks.

To validate the effectiveness of each component in our model, we conducted ablation tests. The results showed that the Autoencoder block significantly improved model efficiency, as evident from the time spent per iteration. For the study of label imbalance problem, Autoencoder and meta-learning strategy had shown

powerful effects through experimental results. In addition, the SE block in the model provided the weight of each brain-region connection, which enhanced model performance.

Our research on feature weights contributes to the significance of implicated brain regions and pathways in Alzheimer's disease. These research findings not only contribute to a deeper understanding of the pathological mechanisms of Alzheimer's disease, but also provide important references for researchers in this field. By providing directional indications of possible abnormalities in certain functional connections, our research provides strong support for biomedical experiments in discovering disease patterns.

We favored of employing small graphs and few gradient descents during the training and testing stages to boost the efficiency of our model and its adaptability to new tasks. In the experiment of the influence of graph size and gradient descent times, we found surprisingly that greatly small graphs and very few gradient descent times did not have a huge impact on the model performance, and the model still brought considerable accuracy results. The extremely small-scale graph simplified the graph structure, and using very few steps mitigated the over-smoothing issue. However, with the enlargement of the graph size and the increase of the number of times for gradient descent, the accuracy rate increased first and then decreased. For larger graphs, the graph information became complicated. And if gradient descent was accomplished too many times, the learning rate should be properly reduced, otherwise the optimal answer may be missed. Of course, to perfectly match the number of times for gradient descent, the selection of learning rate is a tough process that needs constant exploration.

Although our model showed excellent performance in the early diagnosis of Alzheimer's disease, there are still many aspects that may be improved. Firstly, we can explore various feature representations, such as combining image and non-image features as node features. Secondly, only rs-fMRI image data and non-image data such as age were used. Many types of medical images or genetic data are also able to be applied in the field of AD, which can form multimodal studies. However, this may also introduce more data limitation problems, so we believe that continuation of the meta-learning strategy holds promise in addressing these challenges. Moreover, although data acquisition is rather challenging, we still hope to conduct generalization experiments on more databases or clinical data in the future. Additionally, our research focused on the broader three classification task. In this regard, we plan to extend this approach to more nuanced classifications, such as EMCI and LMCI.

References

[1] Kuljeet Singh Anand and Vikas Dhikav. Hippocampus in health and disease: An overview. *Annals of Indian Academy of Neurology*, 15(4):239–246, 2012.

[2] R Cameron Craddock, G Andrew James, Paul E Holtzheimer III, Xiaoping P Hu, and Helen S Mayberg. A whole brain fmri atlas generated via spatially constrained spectral clustering. *Human brain mapping*, 33(8):1914–1928, 2012.

[3] Yuan Luo, Tongtong Sun, Chunchao Ma, Xianchang Zhang, Yong Ji, Xiuwei Fu, and Hongyan Ni. Alterations of brain networks in alzheimer's disease and mild cognitive impairment: a resting state fmri study based on a population-specific brain template. *Neuroscience*, 452:192–207, 2021.

[4] Edmund T Rolls, Marc Joliot, and Nathalie Tzourio-Mazoyer. Implementation of a new parcellation of the orbitofrontal cortex in the automated anatomical labeling atlas. *Neuroimage*, 122:1–5, 2015.

[5] Jinhui Wang, Xindi Wang, Mingrui Xia, Xuhong Liao, Alan Evans, and Yong He. Gretna: a graph theoretical network analysis toolbox for imaging connectomics. *Frontiers in human neuroscience*, 9:386, 2015.

[6] Mingrui Xia, Jinhui Wang, and Yong He. Brainnet viewer: a network visualization tool for human brain connectomics. *PloS one*, 8(7):e68910, 2013.

[7] Liu Yang, Yan Yan, Yonghao Wang, Xiaochen Hu, Jie Lu, Piu Chan, Tianyi Yan, and Ying Han. Gradual disturbances of the amplitude of low-frequency fluctuations (alff) and fractional alff in alzheimer spectrum. *Frontiers in neuroscience*, 12:975, 2018.
